# Association of “hypertriglyceridemic waist” with increased 5-year risk of subclinical atherosclerosis in a multi-ethnic population: a prospective cohort study

**DOI:** 10.1186/s12872-021-01882-1

**Published:** 2021-02-02

**Authors:** Peyman Namdarimoghaddam, Adeleke Fowokan, Karin H. Humphries, G. B. John Mancini, Scott Lear

**Affiliations:** 1 Faculty of Health Sciences, Simon Fraser University, Burnaby, BC V5A 1S6 Canada; 2 Department of Biomedical Physiology and Kinesiology, Simon Fraser University, Burnaby, BC V5A 1S6 Canada; 3 BC Centre for Improved Cardiovascular Health (ICVHealth) at Centre for Health Evaluation and Outcome Sciences (CHEOS), Vancouver, BC V6Z 2K5 Canada; 4 Division of Cardiology, Department of Medicine, University of British Columbia, Vancouver, BC V5Z 1M9 Canada; 5 Division of Cardiology, Providence Health Care, Vancouver, BC V6Z 1Y6 Canada

**Keywords:** Atherosclerotic plaque, Ethnicity, Hypertriglyceridemic waist, Intima-media thickness, Sub-clinical atherosclerosis, Total area, Multi-ethnic, Longitudinal

## Abstract

**Background:**

Hypertriglyceridemic waist (HTGW), which incorporates measures of waist circumference and levels of triglyceride in blood, could act as an early-stage predictor to identify the individuals at high-risk for subclinical atherosclerosis. Previous studies have explored the cross-sectional association between HTGW and atherosclerosis; however, understanding how this association might change over time is necessary. This study will assess the association between HTGW with 5-year subclinical carotid atherosclerosis.

**Methods:**

517 participants of Aboriginal, Chinese, European, and South Asian ethnicities were examined for baseline HTGW and 5-year indices of subclinical atherosclerosis (intima media thickness (mm), total area (mm^2^), and plaque presence). Family history of cardiovascular disease, sociodemographic measures (age, sex, ethnicity, income level, maximum education), and traditional risk factors (systolic blood pressure, smoking status, total cholesterol, high-density lipoprotein cholesterol, body mass index) were incorporated into the models of association. These models used multiple linear regression and logistic regression.

**Results:**

Baseline HTGW phenotype is a statistically significant and clinically meaningful predictor of 5-year intima media thickness (β = 0.08 [0.04, 0.11], *p* < 0.001), total area (β = 0.20 [0.07, 0.33], *p* = 0.002), and plaque presence (OR = 2.17 [1.13, 4.19], *p* = 0.02) compared to the non-HTGW group independent of sociodemographic factors and family history. However, this association is no longer significant after adjusting for the traditional risk factors of atherosclerosis (*p* = 0.27, *p* = 0.45, *p* = 0.66, respectively). Moreover, change in status of HTGW phenotype does not correlate with change in indices of atherosclerosis over 5 years.

**Conclusions:**

Our results suggest that when the traditional risk factors of atherosclerosis are known, HTGW may not offer additional value as a predictor of subclinical atherosclerosis progression over 5 years.

## Background

Cardiovascular diseases (CVDs) are the leading cause of morbidity and mortality globally and the underlying contributor to this burden is mainly atherosclerosis [1]. The pathophysiology of atherosclerosis is well known and progression can be slow, sometimes beginning in childhood and progressing at varying levels of pace into adulthood prior to mediating clinical, ischemic events [2].

A major risk factor for atherosclerosis is visceral obesity that may increase atherosclerosis through metabolic changes which drive the processes of inflammation and fat deposition [3, 4]. Hypertriglyceridemic waist (HTGW) phenotype is a surrogate marker of visceral obesity [5]. Since this phenotype consists of measures of hypertriglyceridemia and elevated waist circumference it can also be used as an inexpensive screening tool to identify individuals at high risk for CVDs. It has been theorized that accumulation of adipose tissue and level of triglyceride present in the blood are chiefly implicated in the risk for atherosclerosis [3]. Though the role of triglyceride levels in the causation of atherosclerosis is still subject of much debate in the scientific community [6]. Nevertheless, HTGW could possibly act as a way to identify individuals at high-risk for subclinical atherosclerosis.

Hypertriglyceridemic waist (HTGW) is primarily a marker of atherogenic metabolic triad which is constituted of hyperinsulinemia, hyperapolipoprotein B, and small LDL particles [7]. The HTGW phenotype has been shown to be a good predictor of increased visceral adiposity [8]. This phenotype has also previously been associated with atherosclerosis [9], abnormal coronary angiography [7, 10, 11], and prospective studies have found HTGW to be a predictor of coronary heart disease [12, 13].

A few studies have previously explored the cross-sectional association between HTGW and atherosclerosis [9]; however, given atherosclerosis is known to develop over time, understanding how this association might change is necessary. Therefore, the purpose of this sub-study was to assess the association between baseline hypertriglyceridemic waist and subclinical carotid atherosclerosis 5 years later using indices of IMT and plaque presence in a multi-ethnic population of men and women with no pre-existing cardiovascular diseases.

## Methods

### Study design

The current investigation is a secondary analysis of the data collected as part of the Multicultural Community Health Assessment Trial (M-CHAT) [14]. The objective of the M-CHAT study was to examine the relationship between obesity and risk for CVD among different ethnic groups. The current investigation curated and analyzed a subset of data from the M-CHAT study to investigate correlations between baseline HTGW and 5-year indices of atherosclerosis.

The M-CHAT study was an observational prospective cohort which followed 517 individuals from Aboriginal, Chinese, European, and South Asian ethnicities for 5 years between 2004 and 2010. Baseline and follow-up data were collected on measures of subclinical atherosclerosis (intima media thickness, presence of plaque and total area), family history of CVD, sociodemographic measures (age, sex, ethnicity, income level, maximum education), and traditional risk factors of atherosclerosis (systolic blood pressure, smoking status, total cholesterol, high-density lipoprotein cholesterol (HDL-C), body mass index (BMI)).

### Setting and participants

The M-CHAT study used a convenience sampling strategy. Participants were enrolled from Aboriginal (reserve and non-reserve), Chinese (China, Hong Kong, Taiwan), European (continental Europe, Ireland, and United Kingdom), and South Asian (Bangladesh, India, Nepal, Pakistan, and Sri Lanka) communities living in, and around, Vancouver, Canada. Participants were recruited by using advertisements in targeted community media (television, radio, print) and posters and brochures at community areas of gatherings and community health fairs. A total of 813 participants between the ages of 30 and 65 years were recruited across different levels of BMI (18.5–24.9, 25–29.9, and > 30 kg/m^2^). The exclusion criteria used in this study were: self-reported history of CVD, recent weight change (greater than 2 kg in 3 months prior to assessment date), use of medications known to affect CVD and type 2 diabetes risk factors (hypoglycemic, antihypertensive, insulin, lipid lowering, or hormone replacement therapy), abusing alcohol or narcotics, diagnosed HIV, metabolic disorders, immune deficiency conditions, or significant amputations/prosthetics.

### Participant assessment

At baseline, participants attended clinics where trained research assistants hired from the target communities assessed them for demographics, anthropometry, health-related behaviours, lipids, and sub-clinical atherosclerosis. Smoking status was determined by a self-report as either never, former (not smoked for 12 months prior to data collection), or current smoker. The maximum education obtained was reported as: less than high school graduate, high school graduate, some post-secondary education, post-secondary degree or diploma, or post-graduate education. Body mass index was calculated as the weight (in kg) divided by the height squared (in meters). Annual income levels were self-reported as < $20,000, $20,000–$30,000, $30,000–$40,000, $50,000–60,000, or > $60,000.

Fasting blood samples were collected and immediately processed for total cholesterol, HDL-C, and triglycerides (TG). All measurements were carried out in the same clinical laboratory with standard enzymatic procedures. Blood pressure was recorded as the average of 5 successive measurements after 10 min of seated rest with an automated oscillometric office blood pressure monitor (VSM MedTech Ltd, Coquitlam, Canada). Blood samples were processed for TG using a standard protocol by the ADVIA 1650 analyzer (Bayer Health Care, Morristown, NI). Those with both elevated TG (males ≥ 2.0 mmol/L; females ≥ 1.5 mmol/L) and elevated waist circumference (males ≥ 90 cm; females ≥ 85 cm) were labelled as HTGW [15].

After approximately 5 years, 517 (of the original 813) participants returned to undergo a second assessment for anthropometry, lipids, and subclinical atherosclerosis. All measurements were taken at the same clinical laboratory using standard procedures. The inter- and intra-assay of this laboratory meet the stringent criteria of the Canadian Reference Foundation Laboratory.

### Carotid assessment

Carotid IMT was measured bilaterally using B-mode ultrasound scans via 7.5 MHz to 10 MHz linear array transducers in the Healthy Heart Clinic at St. Paul’s Hospital in Vancouver, Canada. Scans were then transferred to the Cardiovascular Imaging Research Core Laboratory at Vancouver General Hospital to be digitized for analysis. Each IMT measurement was taken over a 10 mm segment in the far wall of the right and left common coronary carotid artery within 2 cm proximal to the carotid bulb. The regions with the thickest IMT, excluding focal lesions, were measured. Average IMT (mm) was calculated as the mean of the thickness of the right and left carotid arteries. Plaques were identified by at least two observers as points of focally increased thickness in comparison with the IMT thickness to either side of the suspected area. This assessment was conducted in the Cardiovascular Imaging Research Core Lab (CIRCL) in Vancouver, Canada. The intraclass and interobserver correlation values for this method were 0.922 to 0.948 and 0.850 to 0.901, respectively [16]. Total area, which is a superior measure of subclinical atherosclerosis to IMT [16], was calculated as the sum of IMT area (20 mm length × average measured IMT thickness) and the total of all plaque areas (average of all plaque thicknesses × sum of lesion lengths). Thus, average carotid IMT, total area (of plaque and IMT), and plaque presence were used as indices of subclinical atherosclerosis. For repeated measures in the same subject, the accuracy and precision were − 0.001 mm (not significant compared to 0.00 mm) and 0.04 mm, respectively, for IMT and − 0.21 mm^2^ (not significant compared to 0.00 mm^2^) and 3.61 mm^2^, respectively, for total area [16].

### Statistical analysis

Participants with any missing data were excluded from the analysis. All continuous variables were tested for normality. Non-normal variables were reported as median plus interquartile range. Normal variables were reported as mean plus standard deviation. Non-normal variables were log transformed before analysis using the natural logarithm. Categorial variables were reported as numbers and percentages. One-way ANOVA and chi-square tests were used to examine the differences in baseline characteristics between participants in different categories of HTGW phenotype (none, elevated TG only, elevated WC only, HTGW). Independent *t* test and chi-square were used to explore the differences in baseline characteristics between males and females, and the differences in baselines characteristics between participants who completed the 5-year follow up and those who did not. Point-Biserial coefficient and logistic regression were used to assess the bivariate correlation between baseline HTGW categories with 5-year IMT, total area and plaque presence.

Multiple linear regression analysis was used to create models to examine the relationship between baseline HTGW categories with 5-year IMT and total area. Assumptions of multivariate normality, non-multicollinearity, non-autocorrelation, and homoscedasticity were checked. Logistic regression analysis was also used to create other models that examined the association between baseline HTGW categories with 5-year plaque presence. Non-HTGW phenotype was used as the reference group. For both analyses, two models (A and B) were created. Model A adjusted for age, sex, ethnicity, maximum education, income level, and family history of CVD. Model B adjusted for all variables in model A as well as BMI, smoking status, total cholesterol, HDL-C, and systolic blood pressure. Significance level 0.05 was used throughout this study. Statistical analysis was performed using IBM SPSS Statistics version 24.0 (SPSS Inc, Chicago, IL).

## Results

A total of 517 of 813 participants (63%) recruited at baseline completed the 5-year follow-up assessment. The main reasons for not completing the study were lost-to-follow-up in which the participant’s contact information was no longer valid and refusal to participate due to lack of interest. Completers were older (*p* = 0.008), had a lower BMI (*p* = 0.005), waist circumference (*p* < 0.001), less likely to have HTGW (*p* < 0.001), be a current smoker (*p* < 0.001), be Aboriginal (*p* < 0.001), but more likely to have a higher education (*p* < 0.001) and a higher income (*p* < 0.001) than non-completers (Additional file 1: Table S1).

Table 1 outlines the baseline characteristics of the study participants by HTGW components. Significant differences were observed for ethnicity (*p* < 0.001), BMI (*p* < 0.001), total cholesterol (*p* < 0.001), HDL-C (*p* < 0.001), systolic blood pressure (*p* < 0.001) and smoking status (*p* < 0.001) by HTGW phenotype. The prevalence of current smokers was greater in the HTGW group. Baseline IMT and total area also varied significantly by HTGW whereas plaque presence did not.

**Table 1:** Baseline characteristics of the study population stratified by presence of HTGW components

Characteristic	Non-HTGW (Healthy) (n = 312)	Elevated WC (n = 254)	Elevated TG (n = 91)	HTGW (n = 156)	Significance (*p* value)
Age	46.9 ± 8.8	46.9 ± 8.5	46.9 ± 9.1	45.5 ± 8.8	0.36
Sex					
Male	138 (44.2%)	137 (53.9%)	33 (36.3%)	84 (53.8%)	0.006
Ethnicity					< 0.001
Aboriginal	37 (11.9%)	89 (35.0%)	14 (15.4%)	47 (30.1%)	
Chinese	113 (36.2%)	40 (15.7%)	42 (46.2%)	25 (16.0%)	
European	88 (28.2%)	68 (26.8%)	7 (7.7%)	38 (24.4%)	
South Asian	74 (23.7%)	57 (22.4%)	28 (30.8%)	46 (29.5%)	
Family history of CVD present (%)	149 (47.8%)	113 (44.5%)	43 (47.3%)	71 (45.5%)	0.88
Maximum education					0.002
Less than high school	27 (8.7%)	40 (15.7%)	6 (6.6%)	23 (14.7%)	
High school graduate	62 (64.5%)	46 (18.1%)	29 (31.9%)	31 (19.9%)	
Some post-secondary education	35 (11.2%)	48 (18.9%)	10 (11.0%)	24 (15.4%)	
Post-secondary degree/diploma	133 (42.6%)	94 (37.0%)	39 (42.9%)	53 (34.0%)	
Post graduate education	55 (17.6%)	25 (9.8%)	7 (7.7%)	24 (15.4%)	
Annual income level					0.099
< $20,000	36 (11.5%)	36 (14.2%)	14 (15.4%)	24 (15.4%)	
$20,000–$30,000	40 (12.8%)	33 (13.0%)	11 (12.1%)	21 (13.5%)	
$30,000–$40,000	35 (11.2%)	40 (15.7%)	16 (17.6%)	29 (18.6%)	
$40,000–$50,000	40 (12.8%)	25 (9.8%)	9 (9.9%)	25 (16.0%)	
$50,000–$60,000	42 (13.5%)	17 (6.7%)	12 (13.2%)	12 (7.7%)	
> $60,000	115 (36.9%)	95 (37.4%)	28 (30.8%)	44 (28.2%)	
Smoking					< 0.001
Never smoker	215 (68.9%)	140 (55.1%)	68 (74.7%)	79 (50.6%)	
Former smoker	77 (24.7%)	87 (34.3%)	15 (16.5%)	48 (30.8%)	
Current smoker	20 (6.4%)	27 (10.6%)	8 (8.8%)	29 (18.6%)	
Body mass index (kg/m^2^)	24.0 ± 2.8	30.3 ± 4.0	25.1 ± 2.2	31.3 ± 4.4	< 0.001
Total cholesterol (mmol/L)	5.09 ± 0.99	5.00 ± 0.89	5.73 ± 1.07	5.63 ± 0.91	< 0.001
HDL-C (mmol/L)	1.45 ± 0.36	1.26 ± 0.31	1.19 ± 0.35	1.08 ± 0.28	< 0.001
Systolic blood pressure (mm HG)^a^	112 [105, 120]	119 [111, 127]	115 [107, 124]	120 [114, 129]	< 0.001
Intima media thickness (mm)^a^	0.63 [0.58, 0.71]	0.67 [0.60, 0.76]	0.64 [0.59, 0.72]	0.67 [0.60, 0.75]	0.001
Total area (mm^2^)^a^	14.62 [12.20, 21.05]	16.60 [13.10, 25.31]	15.47 [11.90, 24.36]	17.60 [13.50, 23.54]	0.01
Presence of plaque	154 (49.4%)	133 (52.4%)	45 (49.5%)	96 (61.5%)	0.08

Categorical variables presented as n (%). Normally distributed continuous variables presented as mean ± SD

*CVD* cardiovascular disease, *MET* metabolic equivalent, *BMI* body mass index, *HDL-C* high-density lipoprotein cholesterol, *HTGW* hypertriglyceridemic waist, *WC* waist circumference, *TG* triglycerides

Presence of baseline HTGW phenotype was weakly, but positively correlated with 5-year IMT (r_pb_ = 0.11, *p* = 0.02), total area (r_pb_ = 0.13, *p* = 0.00), and plaque presence (2.06 (1.13, 3.74), *p* = 0.02). Additionally, a significant inverse association was observed between the presence of elevated TG phenotype at baseline with the 5-year total area (r_pb_ = − 0.11, *p* = 0.01) (Additional file 1: Table S2). However, this association was not significant when adjusting for sociodemographic and traditional risk factors in Table 2. When adjusting for sociodemographic factors (age, sex, ethnicity, income level, maximum education) and family history in model A, a statistically and clinically significant association was observed between the presence of HTGW phenotype with average IMT (0.08 [0.04, 0.11], *p* < 0.001), total area (0.20 [0.07, 0.33], *p* = 0.002), and plaque presence (OR = 2.17 [1.13, 4.19], *p* = 0.02) compared to the non-HTGW group (Table 2). Presence of elevated WC at baseline was also positively associated with 5-year IMT outcome (0.05 [0.02, 0.09], *p* = 0.002) compared to the non-HTGW group. Upper limits of normal for average IMT and total area are roughly 0.9 mm and 18.3 mm^2^, respectively [16]. When adjusting for traditional risk factors of atherosclerosis (BMI, smoking status, total cholesterol, HDL-C, and systolic blood pressure systolic blood pressure) and sociodemographic factors in model B, we found no significance between elevated WC, elevated TG, and HTGW phenotypes with indices of atherosclerosis, compared to the non-HTGW group. We also investigated the change in standardized β for the variables in each linear regression model (Additional file 1: Table S3) and found that compared to model A, HTGW phenotype was not significant in model B but total cholesterol and systolic blood pressure were significant. Total cholesterol and systolic blood pressure were associated with average IMT (std. β = 0.09, *p* = 0.03; std. β = 0.12, *p* = 0.003), total area (std. β = 0.19, *p* < 0.001; std. β = 0.12, *p* = 0.01), and plaque presence (OR = 1.52, *p* < 0.001; OR = 1.28, *p* = 0.05) in model B. Total cholesterol and systolic blood pressure may be largely mediating the association between elevated WC and HTGW phenotypes with IMT, the effect of HTGW with total area, and the effect of HTGW with plaque presence.

**Table 2:** The association of HTGW phenotype components with 5-year subclinical carotid artery atherosclerosis indices

	Intima media thickness^a^		Total area^a^		Plaque presence^b^	
	β (95% CI)	*p* value	β (95% CI)	*p* value	OR (95% CI)	*p* value
Model A						
Non-HTGW	Reference	Reference	Reference	Reference	Reference	Reference
Elevated WC	0.05 (0.02, 0.09)	0.002	0.05 (− 0.06, 0.16)	0.33	1.03 (0.62, 1.69)	0.92
Elevated TG	0.02 (− 0.03, 0.07)	0.39	− 0.02 (− 0.17, 0.14)	0.85	0.78 (0.40, 1.50)	0.46
HTGW	0.08 (0.04, 0.11)	< 0.001	0.20 (0.07, 0.33)	0.002	2.17 (1.13, 4.19)	0.02
Model B						
Non-HTGW	Reference	Reference	Reference	Reference	Reference	Reference
Elevated WC	0.02 (− 0.02, 0.07)	0.24	0.02 (− 0.11, 0.16)	0.76	0.81 (0.42, 1.56)	0.53
Elevated TG	0.00 (− 0.05, 0.05)	0.94	− 0.12 (− 0.28, 0.04)	0.14	0.51 (0.24, 1.07)	0.08
HTGW	0.03 (− 0.02, 0.08)	0.27	0.06 (− 0.10, 0.22)	0.45	1.21 (0.53, 2.75)	0.66

Outcome variables are in ln(x) form; ^a^multiple linear regression; ^b^ logistic regression; Model A adjusts for age, maximum education, sex, family history, ethnicity, and income level; Model B adjusts for all variables adjusted for in model A plus: BMI, smoking status, total cholesterol, HDL-C, systolic blood pressure; Elevated waist circumference (WC) was ≥ 85 cm in women and ≥ 90 cm in men; Elevated triglycerides (TG) were ≥ 1.5 mmol/L in women and ≥ 2 mmol/L in men; HTGW is the presence of both elevated WC and TG; *BMI* body mass index, *HDL-C* high-density lipoprotein cholesterol, *HTGW* hypertriglyceridemic waist, *WC* waist circumference, *TG* triglycerides

Next, we examined whether there was a correlation between change in HTGW phenotypes with change in the indices of subclinical atherosclerosis over 5 years. The status of change in HTGW phenotypes was categorized into four different groups of (1) Without HTGW at Baseline and Follow-up (N = 376); (2) With HTGW at Baseline and Follow-up (N = 49); (3) Acquired HTGW (N = 50); (4) Reversed HTGW (N = 37). We found that all groups had a significant increase in IMT, total area, and plaque presence in proportions that were similar between groups (IMT *p* = 0.99; total area *p* = 0.35; plaque presence *p* = 0.06) (Table 3). The only exception was the group that Reversed HTGW. This group showed no change in the proportion of participants with plaques present (73.0%). Adjustments for sociodemographic and traditional risk factors did not change these findings (Additional file 1: Table S4 and S5).

**Table 3:** Comparing within- and between- HTGW group differences for atherosclerosis outcome measures without adjusting any covariates

	Without HTGW at baseline and follow-up (N = 376)		With HTGW at baseline and follow-up (N = 49)		Acquired HTGW (N = 50)		Reversed HTGW (N = 37)		Between-group differences
	Baseline	5 year	Baseline	5 year	Baseline	5 year	Baseline	5 year	*p* value
IMT (mm)	0.67 ± 0.12	0.72 ± 0.14*	0.71 ± 0.12	0.77 ± 0.14*	0.65 ± 0.09	0.70 ± 0.11*	0.70 ± 0.13	0.75 ± 0.16*	0.99
Total area (mm^2^)	21.35 ± 16.65	27.82 ± 23.71*	25.64 ± 18.40	35.25 ± 23.92*	17.75 ± 7.45	24.71 ± 14.29*	22.15 ± 13.14	30.54 ± 25.42*	0.35
Plaque presence	200 (53.2%)	252 (67.0%)*	30 (61.2%)	42 (85.7%)*	25 (50.0%)	34 (68.0%)*	27 (73.0%)	27 (73.0%)	0.06

Categorical variables presented as n (%). Continuous variables presented as mean ± SD. Paired-samples *t* test used to test within-group differences for continuous variables. McNemar tests used to test within-group differences for categorical variables. ANCOVA used to test between-group differences for continuous variables. For this test, the follow-up assessment was used as the outcome variable and the baseline variable was controlled for. Chi-square used to test between-group differences for categorical variables. To test between group variables, change in plaque presence was considered in four groups of no change absence of plaque, no change presence of plaque, acquired plaque, plaque reversed. *Marks a significant difference with the corresponding baseline measurement at 0.01 cut-off. Elevated waist circumference (WC) was ≥ 85 cm in women and ≥ 90 cm in men. Elevated triglycerides (TG) were ≥ 1.5 mmol/L in women and ≥ 2 mmol/L in men. HTGW is the presence of both elevated WC and TG. Acquired HTGW refers to those with no HTGW at baseline who tested positive in the follow-up assessment. HTGW reversed, inversely, refers to those with HTGW at baseline who tested negative in the follow-up assessment

Furthermore, the regression models A and B were run a second time for all atherosclerosis indices while controlling for the baseline reading of each index (Table 4). We observed no significant associations between any of the baseline HTGW phenotypes with any of the 5-year indices of atherosclerosis when controlling for the baseline readings of each index.

**Table 4:** Association of HTGW phenotypes with 5-year subclinical atherosclerosis indices while adjusting for each baseline index

	Intima media thickness^a^		Total area^a^		Plaque presence^b^	
	β (95% CI)	*p* value	β (95% CI)	*p* value	OR (95% CI)	*p* value
Model A						
Non-HTGW	Reference	Reference	Reference	Reference	Reference	Reference
Elevated WC	0.02 (0.00, 0.03)	0.10	0.05 (− 0.03, 0.12)	0.22	1.34 (0.71, 2.52)	0.36
Elevated TG	0.02 (− 0.01, 0.05)	0.14	− 0.01 (− 0.11, 0.09)	0.86	0.87 (0.38, 2.00)	0.74
HTGW	0.02 (− 0.01, 0.04)	0.18	0.08 (0.00, 0.16)	0.06	2.04 (0.91, 4.58)	0.09
Model B						
Non-HTGW	Reference	Reference	Reference	Reference	Reference	Reference
Elevated WC	0.01 (− 0.01, 0.03)	0.36	0.02 (− 0.07, 0.11)	0.68	1.33 (0.59, 2.99)	0.49
Elevated TG	0.02 (− 0.01, 0.04)	0.30	− 0.06 (− 0.16, 0.05)	0.30	0.66 (0.26, 1.68)	0.38
HTGW	0.01 (− 0.02, 0.04)	0.58	0.01 (− 0.10, 0.12)	0.82	1.48 (0.53, 4.11)	0.46

Outcome variables are in ln(x) form; ^a^ multiple linear regression; ^b^ logistic regression; Model A adjusts for age, maximum education, sex, family history, ethnicity, and income level; Model B adjusts for all variables adjusted for in model A plus: BMI, smoking status, total cholesterol, HDL-C, systolic blood pressure; Elevated waist circumference (WC) was ≥ 85 cm in women and ≥ 90 cm in men; Elevated triglycerides (TG) were ≥ 1.5 mmol/L in women and ≥ 2 mmol/L in men; HTGW is the presence of both elevated WC and TG; *BMI* body mass index, *HDL-C* high-density lipoprotein cholesterol, *HTGW* hypertriglyceridemic waist, *WC* waist circumference, *TG* triglycerides

## Discussion

This study assessed the relationship between hypertriglyceridemic waist (HTGW) at baseline with 5-year indicators of subclinical atherosclerosis in a multi-ethnic cohort. Our findings indicate that HTGW phenotype is a statistically significant and clinically meaningful predictor of 5-year subclinical atherosclerosis measures of IMT, total area, and plaque presence independent of sociodemographic factors and family history. However, this effect appears to be mediated largely by known risk factors based on our finding that when adjusting for traditional risk factors (total cholesterol and systolic blood pressure), this association was not significant.

Our findings are congruent with the cross-sectional studies conducted in a diverse range of other asymptomatic populations including Caucasian men [17], multi-ethnic populations (Aboriginal, Chinese, European, South Asian) [9], Canadian Cree [18], patients with chronic kidney disease [19], patients living with type 2 diabetes [8], and subjects with cardiometabolic risk factors [20] that found a positive correlation between HTGW and subclinical atherosclerosis. In the total area and plaque presence models, presence of elevated WC or elevated TG alone did not correlate with higher total area or higher proportion with plaque presence. However, the presence of both of these phenotypes (i.e., HTGW phenotype) was correlated with greater total area and a higher proportion with plaque presence. This observation suggests a synergistic elevation of risk. This theory is in line with previous cross-sectional findings in the same multi-ethnic sample that found a stronger correlation between HTGW with subclinical atherosclerosis than between elevated TG and elevated WC individually with subclinical atherosclerosis [9]. Indeed, the HTGW phenotype has been shown to be associated with increased risk of cardiometabolic diseases [21–30]. Additionally, multiple studies have reported an elevated risk of diabetes [31–40] and cardiovascular diseases [12, 41–46] among individuals with HTGW, highlighting the potential value of this phenotype as a marker of CVD risk factors and poor outcomes.

Change in HTGW phenotypes did not correlate with change in indices of subclinical atherosclerosis over 5 years. Hence, acquisition or reversal of HTGW cannot be used as reliable tool to track one’s risk level for atherosclerosis. Within the group that experienced a reversal in their HTGW phenotype, we observed a 5-year halt in the proportion of participants that presented with plaques. This observation indicates that lifestyle measures and reversal of HTGW may be beneficial. However, we did not observe a halt in the progression of IMT or total area in this group. This leads us to hypothesize that the mechanism for increasing IMT and total area might be different from the mechanism for increasing plaque presence which accounts for the difference in associations. This difference in underlying mechanisms would likely be independent of the variables that were adjusted for in our models as our adjustments did not affect this observation. Furthermore, baseline HTGW phenotypes were not associated with 5-year change in indices of atherosclerosis. This finding indicates that whether a patient has any of the HTGW phenotypes does not impact the progression of their atherosclerosis over 5 years.

The strength of our study lies in exploring the relationship between HTGW and subclinical atherosclerosis longitudinally. The ethnic and sociodemographic diversity in our study population allowed us to establish a significantly positive correlation between HTGW with subclinical atherosclerosis that shows independence from income level, maximum education, and ethnicity. Another strength of this study is in using multiple markers of subclinical atherosclerosis (IMT, total area, and presence of plaques) and utilization of carotid ultrasound which have allowed for a robust and direct measure of atherosclerosis, sensitive enough to detect early atherogenesis.

The original study adopted a purposeful sampling strategy which stratified participants by ethnicity and sex and ensured equal representation from each group and may not be representative of the general population. The ethnicities represented in our sample are Aboriginal, Chinese, South Asian, and European communities. Hence, the findings of this paper may not hold valid in other ethnic groups. Furthermore, our assessments did not take into account the fact that categorical assessments of elevated waist circumference may require different cut-offs by ethnicity. Our sample was also limited to individuals between the ages of 30 and 65 years. Hence, the association between HTGW and 5-year subclinical atherosclerosis outcomes in younger and older age groups might need to be further examined. Nevertheless, the age and ethnic groups represented in our sample are those that have been identified at high risk for CVD and should be targeted for screening practices. Our assessment of the association of HTGW phenotypes with the progression of atherosclerosis was limited by the fact that a significant majority of participants already demonstrated evidence of subclinical atherosclerosis at baseline. Future longitudinal studies assessing participants with no evidence of subclinical atherosclerosis at baseline may be able to provide a better assessment of the association between HTGW phenotypes with development and progression of subclinical atherosclerosis. Furthermore, it is possible that the use of more detailed measurements of IMT, including 3D variables, could have detected more subtle findings. However, the use of well validated methods of carotid IMT in this study suggest that use of other well accepted measures of carotid atherosclerotic burden could not have shown different results. As a sub-study, the present investigation consists of secondary analysis. Future studies powered for the outcome may be warranted. Age and gender-based classifications of IMT into normal versus abnormal categories [47] could provide a more clinically meaningful evaluation of participants. However, stratification of participants by age and gender would significantly reduce our statistical power to detect within and between group differences Hence, we used IMT as a continuous variable while adjusting for age and gender in our models. Due to the significant number of non-completers this study is subject to the possibility of type II error. There is also a possibility of residual confounding and random error in this study. Furthermore, while we did not adjust for medication use, only 4.6% of participants were taking a statin at the 5-year follow-up. Therefore, it is unlikely that the use of statins could have had a meaningful impact on our findings.

## Conclusions

Our findings showed that baseline presence of HTGW phenotype in healthy individuals is a significant predictor of 5-year subclinical atherosclerosis independent of sociodemographic factors and family history. However, this effect seems to be largely mediated by known risk factors (total cholesterol and systolic blood pressure) which are often measured at the same time as the elements of HTGW phenotype (TG and WC). Hence, utility of HTGW phenotype as a clinical tool remains unproven. Our results also indicated that the change in presence or absence of HTGW phenotype does not correlate with a change in indices of subclinical atherosclerosis over 5 years. However, in the absence of more long term follow up, data on atherosclerotic burden in other areas such as coronary calcium score, and clinical end points, the utility of monitoring and tracking HTGW cannot be dismissed. Future research should examine the relationship between HTGW phenotype and indices of atherosclerosis among individuals with manifest cardiovascular disease. Another important area for future research is to examine whether there indeed is an alternative mechanism for development of plaque that is distinctively different from IMT and total area. Alternative measurements of hypertriglyceridemic waist or new variables that can be independently measured outside the clinical setting and provide predictive insight, can be highly impactful in identification of at-risk individuals for subclinical atherosclerosis in a way that is cost-effective and adoptable in high numbers.

## Supplementary Information

**Additional file 1:** Supplementary tables.

## Data Availability

The datasets used and/or analysed during the current study are available from the corresponding author on reasonable request.

## Abbreviations

BMI: Body mass index
CVD: Cardiovascular disease
HDL-C: High-density lipoprotein cholesterol
HTGW: Hypertriglyceridemic waist
IMT: Intima media thickness
TG: Triglyceride
WC: Waist circumference
