# Supplementary material for: Association of “hypertriglyceridemic waist” with increased 5-year risk of subclinical atherosclerosis in a multi-ethnic population: a prospective cohort study
Source: BMC Cardiovasc Disord. 2021 Feb 2;21:63. doi: 10.1186/s12872-021-01882-1 (PMC7851930; doi:10.1186/s12872-021-01882-1)
Supplement: Supplementary file 1 — Additional file 1: Supplementary tables. [file 12872_2021_1882_MOESM1_ESM.docx]

**Additional files**

**Table S1**: Baseline characteristics of participants who attended 5-year follow-up (completers) and those who did not (non-completers).

| Characteristic | Non-Completers  (n = 296) | Completers  (n = 517) | Significance (p-value) |
| --- | --- | --- | --- |
| Age | 45.5 ± 8.5 | 47.2 ± 8.9 | 0.008 |
| Sex |  |  |  |
| Male | 136 (45.9%) | 256 (49.5%) | 0.33 |
| Ethnicity |  |  | <0.001 |
| Aboriginal | 115 (38.9%) | 72 (13.9%) |  |
| Chinese | 67 (22.6%) | 153 (29.6%) |  |
| European | 41 (13.9%) | 160 (30.9%) |  |
| South Asian | 73 (24.7%) | 132 (25.5%) |  |
| Family History of CVD Present (%) | 128 (43.2%) | 248 (48.0%) | 0.19 |
| Maximum Education |  |  | <0.001 |
| Less than High School | 50 (16.9%) | 46 (8.9%) |  |
| High School Graduate | 73 (24.7%) | 95 (18.4%) |  |
| Some Post-Secondary Education | 46 (15.5%) | 71 (13.7%) |  |
| Post-Secondary Degree/Diploma | 100 (33.8%) | 219 (42.4%) |  |
| Post Graduate Education | 27 (9.1%) | 84 (16.2%) |  |
| Annual Income Level |  |  | 0.002 |
| < $20,000 | 54 (18.2%) | 56 (10.8%) |  |
| $20,000 - $30,000 | 47 (15.9%) | 58 (11.2%) |  |
| $30,000 - $40,000 | 42 (14.2%) | 78 (15.1%) |  |
| $40,000 - $50,000 | 40 (13.5%) | 59 (11.4%) |  |
| $50,000 - $60,000 | 28 (9.5%) | 55 (10.6%) |  |
| > $60,000 | 79 (26.7%) | 203 (39.3%) |  |
| Smoking |  |  | <0.001 |
| Never Smoker | 166 (56.1%) | 336 (65.0%) |  |
| Former Smoker | 82 (27.7%) | 145 (28.0%) |  |
| Current Smoker | 48 (16.2%) | 36 (7.0%) |  |
| Body Mass Index (kg/m^2^) | 28.1 ± 5.2 | 27.1 ± 4.5 | 0.005 |
| Waist Circumference (cm) | 91.0 ± 13.0 | 87.3 ± 11.5 | <0.001 |
| Total Cholesterol (mmol/L) | 5.20 ± 1.01 | 5.26 ± 0.99 | 0.46 |
| HDL-C (mmol/L) | 1.29 ± 0.35 | 1.29 ± 0.36 | 0.97 |
| Triglycerides (mmol/L)* | 1.39 [0.93, 1.99] | 1.26 [0.90, 1.80] | 0.08 |
| HTGW Phenotype |  |  | <0.001 |
| None | 83 (28.0%) | 229 (44.3%) |  |
| Elevated WC | 106 (35.8%) | 148 (28.6%) |  |
| Elevated TG | 37 (12.5%) | 54 (10.4%) |  |
| HTGW | 70 (23.6%) | 86 (16.6%) |  |
| Systolic Blood Pressure (mm HG)* | 116 [107, 124] | 115 [108, 124] | 0.88 |
| Intima Media Thickness (mm) * | 0.65 [0.59, 0.73] | 0.66 [0.59, 0.74] | 0.87 |
| Total Area (mm^2^) * | 15.10 [12.58, 23.35] | 16.23 [12.43, 23.42] | 0.39 |
| Presence of Plaque | 146 (49.3%) | 282 (54.5%) | 0.15 |

*Categorical variables presented as n (%). Normally distributed continuous variables presented as mean ± SD. *Skewed continuous variables presented as median [25%, 75%]. Completion differences in continuous and categorical variables were explored by independent t-test and Chi-square test, respectively. Elevated waist circumference (WC) was ≥ 85 cm in women and ≥ 90 cm in men. Elevated triglycerides (TG) were ≥ 1.5 mmol/L in women and ≥ 2 mmol/L in men. HTGW is the presence of both elevated WC and TG. CVD: cardiovascular disease; MET: metabolic equivalent of task; BMI: body mass index; HDL-C: high-density lipoprotein cholesterol; HTGW: hypertriglyceridemic waist; WC: waist circumference; TG: triglycerides.*

**Table S2**: Bivariate analysis of HTGW phenotype with 5-year subclinical carotid artery atherosclerosis indices.

|  | Intima Media Thickness | Total Area | Plaque Presence |
| --- | --- | --- | --- |
| Non-HTGW | Reference | Reference | Reference |
| Elevated WC | r_pb_ =0.08, p = 0.09 | r_pb_ =0.05, p = 0.24 | OR = 1.20 (0.77, 1.87), p = 0.43 |
| Elevated TG | r_pb_ =-0.05, p = 0.29 | r_pb_ =-0.11, p = 0.01 | OR = 0.68 (0.37, 1.25), p = 0.22 |
| HTGW | r_pb_ =0.11, p = 0.02 | r_pb_ =0.13, p < 0.001 | OR = 2.06 (1.13, 3.74), p = 0.02 |

r_pb_*: Point-biserial correlation;* OR*: odds ratio from logistic regression; Intima media thickness and total area outcome variables are in ln(x) form*

**Table S3:** The standardized association of baseline characteristics with 5-year subclinical carotid artery atherosclerosis indices.

| Baseline Characteristics | 5-yr Intima Media Thickness (mm) | | 5-yr Total Area (mm^2^) | | 5-yr Plaque Presence ^b^ | |
| --- | --- | --- | --- | --- | --- | --- |
| Model A | Standardized β | p-value | Standardized β | p-value | OR (95% CI) | p-value |
| Phenotype |  |  |  |  |  |  |
| Non-HTGW | Reference | Reference | Reference | Reference | Reference | Reference |
| Elevated WC | 0.14 | 0.00 | 0.04 | 0.33 | 1.03 (0.62, 1.69) | 0.92 |
| Elevated TG | 0.04 | 0.39 | -0.01 | 0.85 | 0.78 (0.40, 1.50) | 0.46 |
| HTGW | 0.16 | 0.00 | 0.13 | 0.00 | 2.17 (1.13, 4.19) | 0.02 |
| Model B |  |  |  |  |  |  |
| Phenotype |  |  |  |  |  |  |
| Non-HTGW | Reference | Reference | Reference | Reference | Reference | Reference |
| Elevated WC | 0.06 | 0.24 | 0.02 | 0.76 | 0.81 (0.42, 1.56) | 0.53 |
| Elevated TG | 0.00 | 0.94 | -0.06 | 0.14 | 0.51 (0.24, 1.07) | 0.08 |
| HTGW | 0.06 | 0.27 | 0.04 | 0.45 | 1.21 (0.53, 2.75) | 0.66 |
| Cholesterol | 0.09 | 0.03 | 0.19 | 0.00 | 1.52 (1.18, 1.94) | 0.00 |
| Blood Pressure | 0.12 | 0.00 | 0.12 | 0.01 | 1.28 (1.00, 1.64) | 0.05 |

*Outcome variables are in ln(x) form; ^a^ multiple linear regression; ^b^ logistic regression; Model A adjusts for age, maximum education, sex, family history, ethnicity, and income level; Model B adjusts for all variables adjusted for in model A plus: BMI, smoking status, total cholesterol, HDL-C, systolic blood pressure; Elevated waist circumference (WC) was ≥ 85 cm in women and ≥ 90 cm in men; Elevated triglycerides (TG) were ≥ 1.5 mmol/L in women and ≥ 2 mmol/L in men; HTGW is the presence of both elevated WC and TG; BMI: body mass index; HDL-C: high-density lipoprotein cholesterol; HTGW: hypertriglyceridemic waist; WC: waist circumference; TG: triglycerides.*

**Table S4**: Degree of change in 5-yr IMT and total area explained by baseline HTGW phenotypes.

| Atherosclerosis Index | 5-yr Change - No adjustments | 5-yr Change - Model A | 5-yr Change - Model B |
| --- | --- | --- | --- |
| Intima Media Thickness* | 0.99 (0.0%) | 0.99 (0.0%) | 0.93 (0.1%) |
| Total Area* | 0.53 (0.4%) | 0.43 (0.5%) | 0.89 (0.1%) |

** Presented as p-values (% variance in outcome explained by HTGW phenotypes). ANCOVA used to test between-group differences. The follow-up assessment was used as the outcome variable and the baseline variable was controlled for. Model A adjusts for sociodemographic factors (age, sex, ethnicity, maximum education, income level) and family history of CVD; Model B adjusts for all variables adjusted for in model A plus traditional risk factors for atherosclerosis (BMI, smoking status, total cholesterol, HDL-C, blood glucose, and systolic blood pressure)*

**Table S5**: Degree of change in 5-yr plaque presence explained by baseline HTGW phenotypes.

| Atherosclerosis Index | 5-yr Change - No adjustments | 5-yr Change - Model A | 5-yr Change - Model B |
| --- | --- | --- | --- |
| Plaque Presence - likelihood ratio | 0.19 | 0.16 | 0.31 |

*Multinomial logistic regression likelihood ratio test presented significance in p-value. Plaque presence reference group is those without plaques in baseline and in follow-up. Model A adjusts for sociodemographic factors (age, sex, ethnicity, maximum education, income level) and family history of CVD; Model B adjusts for all variables adjusted for in model A plus traditional risk factors for atherosclerosis (BMI, smoking status, total cholesterol, HDL-C, blood glucose, and systolic blood pressure).*
